# The Duration of Protection from Azithromycin Against Malaria, Acute Respiratory, Gastrointestinal, and Skin Infections When Given Alongside Seasonal Malaria Chemoprevention: Secondary Analyses of Data from a Clinical Trial in Houndé, Burkina Faso, and Bougouni, Mali

**DOI:** 10.1093/cid/ciaa1905

**Published:** 2021-01-08

**Authors:** Mphatso Dennis Phiri, Matthew Cairns, Issaka Zongo, Frederic Nikiema, Modibo Diarra, Rakiswendé Serge Yerbanga, Amadou Barry, Amadou Tapily, Samba Coumare, Ismaila Thera, Irene Kuepfer, Paul Milligan, Halidou Tinto, Alassane Dicko, Jean Bosco Ouédraogo, Brian Greenwood, Daniel Chandramohan, Issaka Sagara

**Affiliations:** 1 Malaria Epidemiology Group, Malawi-Liverpool-Wellcome Trust Clinical Research Programme, Blantyre, Malawi; 2 Faculty of Epidemiology and Population Health, London School of Hygiene and Tropical Medicine, London, United Kingdom; 3 Le Département Biomédical et de Santé Publique, Institut de Recherche en Sciences de la Santé, Bobo-Dioulasso, Burkina Faso; 4 Malaria Research and Training Center, University of Science, Techniques, and Technologies of Bamako, Bamako, Mali; 5 Faculty of Infectious and Tropical Diseases, London School of Hygiene and Tropical Medicine, London, United Kingdom

**Keywords:** Azithromycin, child mortality, duration of protection, seasonal malaria chemoprevention, Sahel

## Abstract

**Background:**

Mass drug administration (MDA) with azithromycin (AZ) is being considered as a strategy to promote child survival in sub-Saharan Africa, but the mechanism by which AZ reduces mortality is unclear. To better understand the nature and extent of protection provided by AZ, we explored the profile of protection by time since administration, using data from a household-randomized, placebo-controlled trial in Burkina Faso and Mali.

**Methods:**

Between 2014 and 2016, 30 977 children aged 3–59 months received seasonal malaria chemoprevention (SMC) with sulfadoxine-pyrimethamine plus amodiaquine and either AZ or placebo monthly, on 4 occasions each year. Poisson regression with gamma-distributed random effects, accounting for the household randomization and within-individual clustering of illness episodes, was used to compare incidence of prespecified outcomes between SMC+AZ versus SMC+placebo groups in fixed time strata post-treatment. The likelihood ratio test was used to assess evidence for a time-treatment group interaction.

**Results:**

Relative to SMC+placebo, there was no evidence of protection from SMC+AZ against hospital admissions and deaths. Additional protection from SMC+AZ against malaria was confined to the first 2 weeks post-administration (protective efficacy (PE): 24.2% [95% CI: 17.8%, 30.1%]). Gastroenteritis and pneumonia were reduced by 29.9% [21.7; 37.3%], and 34.3% [14.9; 49.3%], respectively, in the first 2 weeks postadministration. Protection against nonmalaria fevers with a skin condition persisted up to 28 days: PE: 46.3% [35.1; 55.6%].

**Conclusions:**

The benefits of AZ-MDA are broad-ranging but short-lived. To maximize impact, timing of AZ-MDA must address the challenge of targeting asynchronous morbidity and mortality peaks from different causes.

## Teaser

Mass administration of azithromycin (AZ-MDA) alongside seasonal malaria chemoprevention in children in Burkina Faso and Mali has broad-ranging but short-lived benefits. To maximize impact, AZ-MDA must address the challenge of targeting asynchronous peaks in morbidity and mortality from different causes.

Despite significant reductions in global child mortality since 1990, 5.3 million under-5-year-old children died in 2018, one-third of these deaths being due to malaria, pneumonia, and diarrhea [1]. Sub-Saharan Africa disproportionately accounted for 52% of these deaths [1]. Given current mortality trends, new tools are urgently required “to end preventable childhood deaths by 2030” (Sustainable Development Goal 3) [1].

Mass drug administration (MDA) of the broad-spectrum macrolide antibiotic azithromycin (AZ) is being considered as a strategy to reduce childhood deaths in sub-Saharan Africa (SSA), with World Health Organization (WHO) guidelines recently published [2]. Several studies, including randomized trials, have reported reductions in childhood mortality associated with AZ-MDA for trachoma control [3, 4]. The MORDOR trial, a large-scale cluster-randomized trial conducted in Niger, Tanzania, and Malawi reported 13.5% fewer deaths in 1–59-month-old children treated with twice yearly AZ [5]. A meta-analysis of 3 cluster-randomized trials of the effect of AZ on childhood deaths reported a 14.4% reduction in communities receiving AZ relative to those who did not receive AZ or received fewer doses [6].

Several critical issues remain if AZ-MDA is to be widely implemented to improve child survival, including understanding the mechanism by which AZ-MDA reduces child mortality. This is needed to understand the optimal timing and dosing frequency for maximum benefit, which may in turn dictate the most appropriate delivery system. There are also concerns about selection for antimicrobial resistance [7–9].

In children, AZ is a safe and efficacious treatment for common bacterial causes of respiratory, gastrointestinal, and skin and soft tissue infections, including *Streptococcus pneumoniae* and *Haemophilus influenza* [10]. Furthermore, AZ is moderately efficacious against *Plasmodium falciparum* [11], which is responsible for 99% of malaria episodes in SSA [12]. It is plausible that AZ reduces childhood mortality by curing infections that commonly cause childhood deaths in the studied areas, including malaria, pneumonia, and infectious diarrhea, as previously suggested [5, 7, 13]. The largest effect in the MORDOR trial was observed among infants, but protection was sustained in older ages in an area of intense, seasonal malaria transmission in Niger, prior to the introduction of Seasonal Malaria Chemoprevention (SMC) in children aged 3–59 months. In addition, the relatively long elimination half-life of AZ (up to 72 hours, after a 3-day course) may provide prophylactic activity for a period after treatment [14, 15].

If AZ-MDA works primarily by short-term cure and prevention, then administration should target periods of maximum childhood mortality. In the Sahel, child deaths increase markedly during a short, intense rainy season, when deaths from malaria peak [16–18]. Monthly administration of AZ, together with SMC, was therefore evaluated over 3 malaria transmission seasons in children aged 3–59 months in Burkina Faso and Mali [19]. There was no reduction in deaths or hospital admissions among children who received SMC plus AZ versus SMC plus placebo, nor was there an important reduction in the overall incidence of clinical malaria [19]. However, reductions of 15% in the incidence of acute lower respiratory tract (ALRI) and gastrointestinal (GI) illnesses, and 40% in nonmalaria fevers with a skin condition were observed [19].

To better understand the extent and potential mechanism of protection provided by AZ, we explored the profile of protection against mortality and hospitalizations, and predefined causes of childhood morbidity (malaria, ALRI, GI, and nonmalaria fevers with a skin condition) using data from the above trial of AZ co-administered with SMC; the ‘SMC+AZ trial’.

## METHODS

### Study Setting

The SMC+AZ trial was a household-randomized, placebo-controlled trial conducted in Houndé district, Burkina Faso, and Bougouni district, Mali, between August 2014 and December 2016. Detailed methods are reported elsewhere [19]. Briefly, children resident in the study area were enumerated in a census in early 2014. Children aged 3–59 months at the first SMC cycle in August 2014 received a 3-day SMC course with sulfadoxine pyrimethamine (SP) plus amodiaquine (AQ) and either AZ (SMC+AZ), or a matching placebo (SMC+placebo), monthly up to 4 times each year as directly-observed therapy. At the start of the rainy seasons in 2015 and 2016, newly eligible children aged 3–59 months (births or migrations into the study area) were enumerated and included if caregivers gave consent. Children who reached age 5 years during the study were not treated in subsequent intervention years. A total of 30 977 children (~20 000 each year) contributed person-time at risk over the study period.

### Definition of Outcomes

Mortality, hospitalization, and morbidity were recorded passively at hospitals and health facilities in the study area, with verbal autopsy used to investigate deaths outside health facilities. The primary outcome was death or hospital admission for ≥24 hours not due to trauma or elective surgery during the intervention period (defined as the period from administration of the first SMC dose until 30 days after administration of the last SMC course, each year). Trained health workers systematically diagnosed morbidity episodes recorded passively among children attending outpatient clinics. In Mali only, community health workers also confirmed, treated, and documented malaria episodes. Malaria was defined as reported fever within 24 hours or measured temperature ≥37.5°C plus a positive histidine-rich protein 2 (HRP-2)-based rapid diagnostic test (RDT) or blood smear confirming *P. falciparum* infection. Blood smears for RDT quality control were taken from a systematic sample of study children presenting at study clinics (1 day/week in Burkina Faso, and 1 week/month in Mali). Acute lower respiratory tract (ALRI) and gastrointestinal illnesses (GI) were diagnosed using WHO standard case definitions [20]. Clinic attendances for nonmalaria fevers (NMFs) for which a skin condition was the primary diagnosis, hereafter “NMFs with a skin condition,” were identified by review of case report forms for all NMF episodes by 2 independent clinicians [19]. For these analyses, we excluded ALRI episodes from Burkina Faso as the number of events was too low to support an analysis by time since treatment.

To determine if the effect of AZ changed over time, the incidence of 5 outcomes was compared between study groups in different time periods post-treatment: 1) deaths or hospital admissions not due to trauma or elective surgery; 2) RDT-confirmed clinical malaria, 3) ALRI, 4) GI, and 5) NMF with a skin condition. For each child treated with at least 1 dose of SMC+AZ or SMC+placebo (ie, a modified intention-to-treat analysis), person-time at risk was calculated from the first dose of SMC+AZ or SMC+placebo each month until the next monthly dose, or the end of the intervention period that year. Person-time was censored if loss to follow-up, death, or voluntary withdrawal occurred. Lexis expansion was used to further stratify follow-up time after each SMC course for each child into 7-day time strata. To avoid double counting disease episodes that resulted in multiple healthcare contacts, morbidity episodes documented within 7 days of a previous episode of the same type were not counted. No adjustment was made to the person-time at risk [21].

### Statistical Analysis

Poisson regression models, with a gamma-distributed random effect to account for the household-randomized design and within-individual clustering of morbidity episodes, were used to obtain time stratum-specific incidence rate ratios (IRR) for each outcome, comparing SMC+AZ versus SMC+placebo groups [22]. The likelihood ratio test (LRT) was used to formally assess evidence for an interaction between treatment group and time stratum. Protective efficacy (PE) was 1-IRR. To improve precision, person-time and events within equivalent time strata following separate monthly treatments (eg, 0–7 days post first SMC, 0–7 days post second SMC, etc.) were pooled, with incidence rate calculated as number of events divided by total person-time at risk. This assumes the benefit of AZ was consistent after different monthly courses, an assumption we explored by examining evidence for effect modification by course. The primary analysis for each outcome was adjusted for country a priori, as in the primary trial. Findings remained unchanged when also adjusted for age and/or sex, so PEs are presented from analyses adjusted only for country. All analyses were performed in Stata 15 SE® (StataCorp, College Station, Texas).

## RESULTS

### Characteristics of Treated Children, Treatment Adherence, and Follow-Up

Overall, 30 629 (98.9%) of 30 977 study children received at least 1 dose of study medication. All results hereafter refer to these “treated children,” who were similar across SMC+AZ and SMC+placebo groups in terms of sex, age at enrollment, and country (Table 1). Adherence to the SMC schedule was similar between groups: >65% received all 4 monthly cycles each year and >84% received at least 3 cycles [19]. Adherence to the 3-day regimen in children who received the first dose was >91% at all SMC contacts, and >96% at 9 of the 12 contacts (Supplementary Table 1). Person-time at risk accrued was similar between SMC+AZ and SMC+placebo groups: 9,083.4 and 9,064.3 person-years, respectively.

**Table 1.** Characteristics of Children by Study Group

Characteristic	SMC plus placebo	SMC plus AZ
**Total, n (%)**	15 339 (50.1)	15 290 (49.9)
**Country, n (%)**		
Burkina Faso	7540 (49.2)	7612 (49.8)
Mali	7799 (50.8)	7678 (50.2)
**Sex, n (%)**		
Male	7688 (50.1)	7700 (50.4)
Female	7358 (48.0)	7287 (47.7)
Missing	293 (1.91)	303 (1.98)
**Age (y) at first SMC, n (%)**		
<1	4511 (29.4)	4550 (29.8)
1	3378 (22.0)	3392 (22.2)
2	2705 (17.6)	2644 (17.3)
3	2510 (16.4)	2426 (15.9)
4	2235 (14.6)	2278 (14.9)
**Mean age at all SMC contacts, y (SD**)	2.70 (1.35)	2.69 (1.35)

Abbreviations: AZ, azithromycin; SD, standard deviation; SMC, seasonal malaria chemoprevention.

### Incidence and Protective Efficacy of AZ by Time Since Treatment

#### Hospital Admissions and Deaths

**Figure 1.**
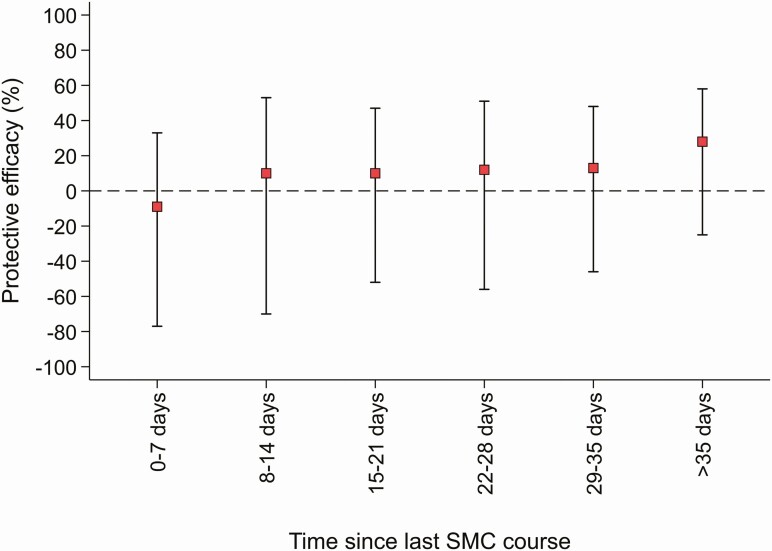
Protective efficacy of azithromycin against the primary outcome (deaths and hospital admissions) by time since most recent seasonal malaria chemoprevention (SMC) course. Protective Efficacy (%) was calculated as (1—Incidence Rate Ratio) X 100. Incidence Rate was calculated as the total number of events divided by total person-years at risk. For each time stratum, incidence rate was calculated as number of events divided by person-years at risk. Rate ratios compare SMC+Azithromycin versus SMC+placebo groups. Poisson regression models, with a gamma distributed random effect to account for the household randomisation and within-individual clustering of morbidity episodes. Models were adjusted for study country only. Red squares are point estimates, solid vertical lines indicate 95% confidence intervals. Dashed horizontal line indicates no protective efficacy of azithromycin.

Overall, 151 and 170 hospital admissions and deaths occurred during the intervention periods in the SMC+AZ and SMC+placebo groups: 16.6 [95% Confidence Interval (CI): 14.2; 19.6] and 18.8 [16.0; 22.2] episodes per 1000 person-years at risk (PYAR), respectively (Table 2). Incidence rates increased by time since treatment in both groups as protection from monthly SMC waned, but the effect of AZ did not differ with time since treatment: likelihood ratio test (LRT) for time-treatment group interaction, *P* = .94. No benefit of AZ was seen in any time stratum post-treatment (Table 2, Figure 1).

**Table 2.** Incidence of the Primary Outcome (Death and Hospital Admissions Not Due to Trauma or Elective Surgery) by Time Since the Most Recent SMC Treatment

	SMC plus Placebo		SMC plus Azithromycin			
Days since last SMC	No. of events (PYAR	Rate per 1000 person-years at risk (95% CI)	No. of events (PYAR)	Rate per 1000 person-years at risk (95% CI)	Rate Ratio^a^ (95% CI)	LRT^b^*P*-value
0–7	32 (2044.3)	15.7 (11.2, 22.5)	35 (2051.4)	17.1 (12.3, 24.4)	1.09 (0.67, 1.77)	
8–14	20 (2030.3)	9.85 (6.45, 15.8)	18 (2036.9)	8.84 (5.39, 15.6)	0.90 (0.47, 1.70)	
15–21	30 (1983.8)	15.1 (10.6, 22.4)	27 (1991)	13.6 (9.42, 20.3)	0.90 (0.53, 1.52)	
22–28	25 (1805.4)	13.8 (9.48, 21.1)	22 (1814.5)	12.1 (8.11, 19.0)	0.88 (0.49, 1.56)	
29–35	31 (701.8)	44.2 (31.4, 64.1)	27 (704.7)	38.3 (26.6, 57.2)	0.87 (0.52, 1.46)	
>35	32 (498.8)	64.2 (45.0, 94.8)	22 (484.9)	45.4 (30.3, 71.1)	0.72 (0.42, 1.25)	.94

Abbreviations: CI, confidence interval; LRT, Likelihood ration test; PYAR, person-years at risk; SMC, seasonal malaria chemoprevention.

^a^For each time stratum, incidence rate was calculated as number of events divided by person-years at risk. Rate ratios compare SMC+Azithromycin versus SMC+placebo groups. Poisson regression models, with a gamma distributed random effect to account for the household randomisation and within-individual clustering of morbidity episodes. Models were adjusted for study country only.

^b^Likelihood ratio test comparing models with and without an interaction between treatment group and time since treatment.

### RDT-Confirmed Clinical Malaria

**Figure 2.**
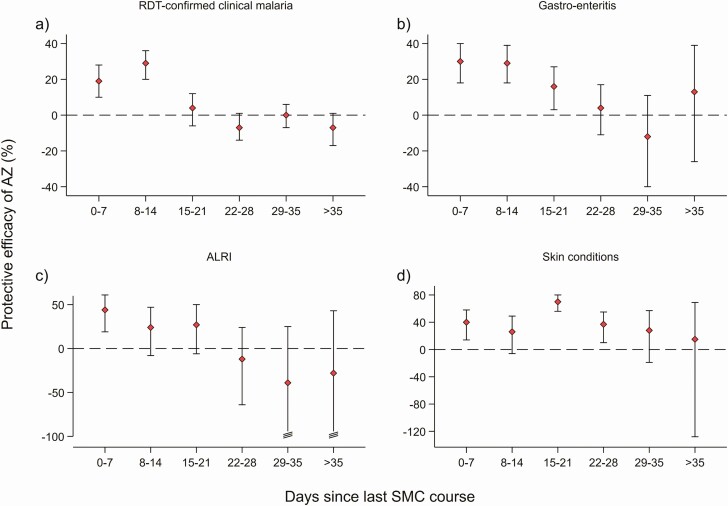
Protective efficacy of azithromycin against predefined secondary outcomes by time since most recent SMC course: (A) RDT-confirmed clinical malaria; (B) Gastroenteritis; (C) Acute lower respiratory tract infections; and (D) Non-malaria fevers with a skin condition. For each time stratum, incidence rate was calculated as number of events divided by person-years at risk. Rate ratios compare SMC+Azithromycin versus SMC+placebo groups. Poisson regression models, with a gamma distributed random effect to account for the household randomisation and within-individual clustering of morbidity episodes. Models were adjusted for study country only. Red squares are point estimates, solid vertical lines indicate 95% confidence intervals. Dashed horizontal line indicates no protective efficacy of azithromycin. For clarity of presentation the Y-axis is truncated at -100 for ALRI. The lower limit of the CIs for 29–35 days and >35 days extend to –156% and -188%, respectively. Abbreviations: ALRI, acute lower respiratory tract infections; AZ, azithromycin; RDT, rapid diagnosic test; SMC seasonal malaria chemoprevention.

In the SMC+AZ and SMC+placebo groups, 6963 and 7204 RDT-confirmed malaria episodes were recorded, respectively, 756.2 [733.8; 779.2] and 777.6 [755; 800.9] episodes per 1000 PYAR (Table 2). The effect of AZ differed with time since treatment (LRT, *P* < .001). Incidence was lower in weeks 1 and 2 post-treatment in SMC+AZ than SMC+placebo recipients, protective efficacies (PE): 19.2% [9.59; 27.9%, *P* < .001] and 28.6% [20.1; 36.1%, *P* < .001], respectively, and 24.2% [17.8%, 30.1%, *P* < .001], in the first 2 weeks overall. There was no evidence of protection beyond week 2. In weeks 4 and 6, incidence was higher in the SMC+AZ group, although 95% CIs for protective efficacy overlapped unity in both cases (Table 3, Figure 2).

**Table 3.** Incidence of Secondary Outcomes by Time Since the Most Recent SMC Treatment

	SMC plus placebo		SMC plus azithromycin			
Days since last SMC	No. of events (PYAR)	Rate per 1000 person-years at risk (95% CI)	No. of events (PYAR)	Rate per 1000 person-years at risk (95% CI)	Rate Ratio^a^ (95% CI)	LRT^b^*P*-value
**RDT-confirmed** **Malaria**						
0–7	704 (2044.3)	344.4 (318.7, 372.7)	565 (2051.4)	275.4 (253.1, 300.3)	0.81 (0.72, 0.90)	
8–14	776 (2030.3)	382.2 (353.9, 413.5)	552 (2036.9)	271.0 (248.6, 296.1)	0.71 (0.64, 0.80)	
15–21	956 (1983.8)	481.9 (450.8, 515.8)	918 (1991)	461.1 (430.4, 494.6)	0.96 (0.88, 1.06)	
22–28	1640 (1805.4)	908.4 (862.3, 957.7)	1743 (1814.5)	960.6 (911.7, 1012.9)	1.07 (0.99, 1.14)	
29–35	1851 (701.8)	2637.3 (2518.8, 2763.2)	1851 (704.7)	2626.8 (2504.1, 2757.2)	1.00 (0.94, 1.07)	
>35	1277 (498.8)	2560.2 (2424.3, 2705.9)	1334 (484.9)	2751.0 (2603.9, 2908.6)	1.07 (0.99, 1.17)	<.001
**Gastroenteritis**						
0–7	394 (2044.3)	192.7 (174.2, 213.8)	273 (2051.4)	133.1 (117.6, 151.2)	0.70 (0.60, 0.82)	
8–14	469 (2030.3)	231.0 (210.5, 254.1)	327 (2036.9)	160.5 (143.8, 179.9)	0.71 (0.61, 0.82)	
15–21	443 (1983.8)	223.3 (202.4, 247.0)	368 (1991)	184.8 (166.4, 205.9)	0.84 (0.73, 0.97)	
22–28	390 (1805.4)	216.0 (195.0, 240.1)	370 (1814.5)	203.9 (183.8, 226.9)	0.96 (0.83, 1.11)	
29–35	145 (701.8)	206.6 (175.7, 244.6)	161 (704.7)	228.5 (195.0, 269.5)	1.12 (0.89, 1.40)	
>35	65 (498.8)	130.3 (102.5, 168.3)	55 (484.9)	113.4 (87.5, 149.8)	0.87 (0.61, 1.26)	<.001
**Acute Lower Respiratory Tract Infection**						
0–7	88 (2044.3)	84.5 (68.4, 105.7)	50 (2051.4)	48.8 (37.0, 65.6)	0.56 (0.39, 0.81)	
8–14	76 (2030.3)	73.0 (57.8, 93.5)	58 (2036.9)	56.6 (43.1, 75.9)	0.76 (0.53, 1.08)	
15–21	67 (1983.8)	64.5 (50.6, 83.5)	49 (1991)	47.9 (35.4, 66.3)	0.73 (0.50, 1.06)	
22–28	53 (1805.4)	56.9 (42.6, 77.7)	60 (1814.5)	65.2 (50.9, 85.0)	1.12 (0.76, 1.64)	
29–35	18 (701.8)	56.2 (35.1, 95.9)	25 (704.7)	79.8 (54.8, 120.8)	1.39 (0.75, 2.56)	
>35	11 (498.8)	39.7 (22.6, 76.9)	13 (484.9)	51.7 (30.6, 94.6)	1.28 (0.57, 2.88)	.033
**Non-malaria fevers with a skin condition**						
0–7	81 (2044.3)	39.6 (31.9, 49.3)	48 (2051.4)	23.4 (17.6, 31.0)	0.60 (0.42, 0.86)	
8–14	73 (2030.3)	36.0 (28.6, 45.2)	53 (2036.9)	26.0 (19.9, 34.1)	0.74 (0.51, 1.06)	
15–21	116 (1983.8)	58.5 (48.7, 70.1)	34 (1991)	17.1 (12.2, 23.9)	0.30 (0.20, 0.44)	
22–28	85 (1805.4)	47.1 (38.1, 58.2)	53 (1814.5)	29.2 (22.3, 38.2)	0.63 (0.45, 0.90)	
29–35	37 (701.8)	52.7 (38.2, 72.8)	26 (704.7)	36.9 (25.1, 54.2)	0.72 (0.43, 1.19)	
>35	9 (498.8)	18.0 (9.39, 34.7)	7 (484.9)	14.4 (6.88, 30.3)	0.85 (0.31, 2.28)	.006

Abbreviations: CI, confidence interval; LRT, likelihood ratio test; PYAR, person-years at risk; RDT, rapid diagnostic test; SMC, seasonal malaria chemoprevention.

^a^For each time stratum, incidence rate was calculated as number of events divided by person-years at risk. Rate ratios compare SMC+Azithromycin versus SMC+placebo groups. Poisson regression models, with a gamma distributed random effect to account for the household randomisation and within-individual clustering of morbidity episodes. Models were adjusted for study country only.

^b^Likelihood ratio test comparing models with and without an interaction between treatment group and time since treatment.

For the 1444 RDT-confirmed malaria episodes with a corresponding blood slide, the slide was positive for 712 cases (RDT positive predictive value, PPV: 49.3% [46.7, 51.9]. However, the PPV of a positive RDT changed sharply over time since SMC, ranging from 18.4% [14.6, 22.7] in the first 2 weeks to 74.4% [68.3, 79.8] beyond 35 days (Supplementary Table 2). When restricted to the sub-set of malaria episodes confirmed by microscopy, the short-term benefit of AZ on malaria was no longer observed (Supplementary Table 3, Supplementary Figure 1, Supplementary Figure 2).

### Gastrointestinal Illness (GI)

In the SMC+AZ and SMC+placebo groups, 154 and 1906 GI episodes were recorded, respectively, 170.9 [161.5; 180.8] and 207.5 [197.3; 218.3] episodes per 1000 PYAR (Table 2). The effect of AZ varied with time post-treatment, LRT *P* = .001. Compared to SMC+placebo, GI incidence was 30% lower in SMC+AZ recipients in each of weeks 1 and 2 post-treatment, (overall PE for the first two weeks: 29.9% [21.7; 37.3%, *P* < .001]), and 16% lower in week 3 (PE: 16% [3.1; 27.2%, *P* = .02] (Table 3, Figure 2).

### Acute Lower Respiratory Tract Infections (ALRI)

In Mali, 568 ALRI episodes were recorded among treated children: 255 and 313 in SMC+AZ and SMC+placebo groups: 56 [48.5; 64.6] and 68.3 [60.2; 77.6] episodes per 1000 PYAR, respectively. The effect of AZ varied with time post-treatment, LRT *P* = .03 (Table 2). ALRI incidence was 44% lower in the SMC+AZ group than in the SMC+placebo group in the first week post-treatment, PE: 44.0% [20.2; 60.7], *P* = .002. There was no evidence of a protective effect of AZ beyond the first week post-treatment. Point estimates were consistent with a modest benefit in weeks 2 and 3, and with negative protective efficacy in week 4, but confidence intervals were wide. (Table 3, Figure 2)

### Non-Malaria Fevers With a Skin Condition

Overall, 221 vs 421 episodes of NMFs with a skin condition were recorded in SMC+AZ vs SMC+placebo groups, thus 24.3 [21.3; 27.8] and 44.2 [40.1; 48.8] episodes per 1000 PYAR, respectively. The effect of AZ varied with time post-treatment, LRT *P* = .006. (Table 3 and Figure 2). Incidence of NMFs with a skin condition was lower among SMC+AZ-treated children in the first month post-treatment, with an overall PE during this period of 46.3% [35.1; 55.6%], *P* < .001 (Table 3, Figure 2).

## DISCUSSION

This study evaluated the extent and duration of protection from AZ when co-administered with SMC in 3–59-month-old children in Burkina Faso and Mali. Consistent with the overall findings of the trial, there was no indication of even a transient benefit from AZ against hospital admissions or deaths. The modest overall protective efficacy of AZ against GI and ALRI observed in the trial, approximately 15%, was driven by a high initial level of protection that waned over a period of a few weeks. The higher protection against NMFs with a skin condition lasted somewhat longer, but was still time-limited.

The apparent additional benefit of AZ against malaria in the first 2 weeks after SMC was surprising, because SP+AQ provides a high level of protection against *P. falciparum* for up to 4 weeks post-treatment, which then wanes rapidly [23]. This may reflect false positives arising from use of RDTs to confirm malaria, since *P. falciparum* antigens may persist for a few weeks even if infections are cleared by SMC [24]. It is possible that AZ reduced the incidence of fevers due to causes other than malaria in the weeks immediately after SMC, and that this—rather than a direct effect of AZ on malaria—led to fewer episodes of malaria being diagnosed by RDT in the SMC+AZ group. This is plausible given the short-term impact of AZ on several other causes of fever in this study, and is supported by our sensitivity analysis restricting to the sub-set of malaria cases confirmed by microscopy. However, it is uncertain if this applies to all 14 000 RDT-confirmed malaria episodes, as most did not have an accompanying blood smear. Alternatively, this may be partly explained by the very high incidence rates in the 2 study areas, ie, that even very high SMC efficacy allows some malaria cases to occur, allowing AZ to improve protection. If the impact of AZ on malaria is genuine, as observed elsewhere [25, 26], then this suggests either improved cure of existing infections, a short-lived contribution to post-treatment prophylaxis, or both [15, 27–29].

There was strong evidence of protection against GI and NMFs with a skin condition for the first 3 and 4 weeks post-treatment, respectively. Point estimates for ALRI were also compatible with protection over a similar period, although confidence intervals were wider, reflecting the smaller number of episodes [30, 31]. The addition of AZ to chloroquine for treatment of uncomplicated malaria reduced ALRI and GI incidence in Malawian children [32]. AZ was also associated with reduced pathogenic gut bacteria, and altered gut microbiome structures in Indian and Nigerien children, respectively [33–35].

The finding of no short-term benefit of AZ against hospital admissions and deaths, in contrast to the MORDOR trial [36, 37], is logical given the slightly higher overall incidence rate of the primary outcome in SMC+AZ recipients in the main trial. Notwithstanding, it is plausible, based on our findings, that main impact of AZ is intermittent clearance of pathogenic organisms, with short-term prevention of reinfection while AZ blood concentrations remain sufficiently high, ie, similar to SMC and other malaria chemopreventive approaches [23, 38]. If true, the impact of AZ-MDA would be maximized when administered around the time of peak morbidity and mortality. However, since the relative importance of cure and prophylaxis from AZ remains unclear, it is not obvious how to balance the risks of giving AZ too early, and leaving children exposed to reinfection when prophylaxis wanes, versus giving AZ too late, and children suffering morbidity and mortality from infections acquired prior to administration. Furthermore, unlike SMC, AZ is not targeted at a specific pathogen, and thus optimum administration requires targeting asynchronous peaks in morbidity and mortality from multiple causes.

Strengths of this study include the large number of events and person-time, which allowed estimation of PEs with reasonable precision in relatively small time strata, particularly for malaria and GI. Results were similar with 5- or 10-day strata, so it is unlikely that using week-long strata affected our findings or interpretation. All doses of study medication were directly-observed and documented using tablet computers, ensuring high accuracy for recorded treatment dates. Bias due to differential reporting and/or diagnoses between treatment groups is unlikely, given both participant and observer blinding. Key limitations are that our analysis was, by design, restricted to children who received at least 1 dose of study medication, which could introduce bias if untreated children differed systematically from treated children [39]. However, almost all study children contributed to these analyses, and the overall IRRs of pooled person-time at risk in treated children were similar to estimates from the intention-to-treat cohort [19]. A further limitation is the lack of confirmatory microbiological data for ALRI, GI, and NMFs with a skin condition, which precluded pathogen-specific analyses.

The duration of protection estimated in our household-randomized study, with drug administration restricted to children below 5 years of age, may be shorter than in a community-wide implementation in which all ages groups are treated. The former situation could allow reinfection from nontreated individuals sooner after treatment [40]. Consequently, our findings may not be fully generalizable to community-wide AZ-MDA.

Although a limitation for our study, the finding that the positive predictive value of RDTs was low immediately after SMC administration may have important implications for routine evaluation of SMC programmes. In high transmission areas, persistent HRP-2 antigenemia for some weeks after treatment, combined with the high curative efficacy of SP+AQ, will inevitably lead to false positives. While RDTs are an essential tool for case-management, routine data based only on RDTs may overestimate the burden of malaria occurring in the context of SMC, potentially causing false alarm about low effectiveness of SP+AQ. Blood smears, which have higher specificity, are needed to monitor SMC.

The benefits of AZ administration appear to be short-lived, consistent with the effect being due to cure of existing pathogens and short-term prophylaxis. To maximize impact, further research may be needed where AZ-MDA is being considered to ascertain the optimal timing of administration so as to target periods of peak disease risk from AZ-susceptible pathogens.

## Supplementary Data

Supplementary materials are available at *Clinical Infectious Diseases* online. Consisting of data provided by the authors to benefit the reader, the posted materials are not copyedited and are the sole responsibility of the authors, so questions or comments should be addressed to the corresponding author.

ciaa1905_suppl_Supplementary_Materials

## Notes

**Author Contributions.** All authors confirmed they have contributed to the intellectual content of this paper and have met the following 4 requirements: (a) significant contributions to the conception and design, acquisition of data, or analysis and interpretation of data; (b) drafting or revising the article for intellectual content; (c) final approval of the published article; and (d) agreement to be accountable for all aspects of the article thus ensuring that questions related to the accuracy or integrity of any part of the article are appropriately investigated and resolved.

***Acknowledgments.*** The authors thank the trial steering committee (Feiko ter Kuile [chair], Kalifa Bojang, Kojo Koram, David Mabey, Morven Roberts, and Mahamadou Thera) and the data and safety monitoring board (Blaise Genton [chair], Cheick Oumar Coulibaly, Umberto D’Alessandro, and Francesca Little) for their assistance with the trial, the Ministry of Health staff in the Bougouni and Houndé districts for their assistance; and all the caretakers and children for their participation.

***Financial support.*** This work was supported by the Joint Global Health Trials scheme, which is funded by the U.K. Medical Research Council, Department for International Development, National Institute for Health Research, and the Wellcome Trust [grant number: MR/K007319/1]; a Wellcome Trust Master’s Fellowship in Public Health and Tropical Medicine, grant number: 205311/Z/16/Z to M.P. who undertook some of this work during an MSc in Epidemiology at LSHTM; and an award jointly funded by the UK Medical Research Council (MRC) and the UK Department for International Development (DFID) under the MRC/DFID Concordat agreement, which is also part of the EDCTP2 programme supported by the European Union, grant number: MR/R010161/1, which supported MC.

***Potential conflicts of interest.*** The authors: No reported conflicts of interest. All authors have submitted the ICMJE Form for Disclosure of Potential Conflicts of Interest.
