# Supplementary material for: The Duration of Protection from Azithromycin Against Malaria, Acute Respiratory, Gastrointestinal, and Skin Infections When Given Alongside Seasonal Malaria Chemoprevention: Secondary Analyses of Data from a Clinical Trial in Houndé, Burkina Faso, and Bougouni, Mali
Source: Clin Infect Dis. 2021 Jan 8;73(7):e2379–86. doi: 10.1093/cid/ciaa1905 (PMC8492219; doi:10.1093/cid/ciaa1905)
Supplement: ciaa1905_suppl_Supplementary_Materials [file ciaa1905_suppl_supplementary_materials.docx]

**Supplementary Table 1**. Number and percentage of children who received all three daily doses of seasonal malaria chemoprevention and either azithromycin or placebo, among those who received the first dose of each monthly course.

| **Year** | **SMC ^a^ course** | **Burkina Faso** |  |  |  | **Mali** |  |  |  |
| --- | --- | --- | --- | --- | --- | --- | --- | --- | --- |
|  |  | **SMC+Placebo** |  | **SMC+AZ ^b^** |  | **SMC+Placebo** |  | **SMC+AZ ^b^** |  |
|  |  | n/N | % | n/N | % | n/N | % | n/N | % |
| 2014 | 1 | 4051/4279 | 94.7 | 4143/4361 | 95.0 | 3955/4336 | 91.2 | 3818/4171 | 91.5 |
|  | 2 | 4205/4344 | 96.8 | 4249/4386 | 96.9 | 4023/4308 | 93.4 | 3890/4193 | 92.8 |
|  | 3 | 4205/4315 | 97.5 | 4305/4411 | 97.6 | 4143/4394 | 94.3 | 4101/4302 | 95.3 |
|  | 4 | 4429/4513 | 98.1 | 4499/4601 | 97.8 | 4287/4455 | 96.2 | 4175/4337 | 96.3 |
|  |  |  |  |  |  |  |  |  |  |
| 2015 | 1 | 4350/4394 | 99.0 | 4450/4487 | 99.2 | 4424/4599 | 96.2 | 4333/4489 | 96.5 |
|  | 2 | 4515/4546 | 99.3 | 4590/4627 | 99.2 | 4631/4734 | 97.8 | 4589/4670 | 98.3 |
|  | 3 | 4473/4505 | 99.3 | 4529/4557 | 99.4 | 4592/4691 | 97.9 | 4539/4654 | 97.5 |
|  | 4 | 4488/4507 | 99.6 | 4531/4544 | 99.7 | 4471/4509 | 99.2 | 4484/4509 | 99.5 |
|  |  |  |  |  |  |  |  |  |  |
| 2016 | 1 | 4443/4478 | 99.2 | 4519/4554 | 99.2 | 4334/4478 | 96.8 | 4285/4417 | 97.0 |
|  | 2 | 4591/4606 | 99.7 | 4609/4625 | 99.7 | 4727/4762 | 99.3 | 4668/4693 | 99.5 |
|  | 3 | 4598/4608 | 99.8 | 4645/4655 | 99.8 | 4439/4484 | 99.0 | 4461/4503 | 99.1 |
|  | 4 | 4622/4629 | 99.9 | 4644/4662 | 99.6 | 4547/4588 | 99.1 | 4503/4561 | 98.7 |

**Footnotes**

^a^ SMC: seasonal malaria chemoprevention

^b^ AZ: azithromycin**Supplementary Table 2.** Positive Predictive Value of the malaria rapid diagnostic test by time since most recent SMC, for the sub-set of children with both a blood slide and RDT result

| **Days since last SMC ^a^** | **RDT^b^-confirmed episodes with a slide result** | **Blood slide negative** | **Blood slide positive** | **Positive Predictive Value (95% CI)** |
| --- | --- | --- | --- | --- |
| ≤ 7 | 173 | 122 | 51 | 29.5 (22.8, 36.9) |
| 8-14 | 202 | 184 | 18 | 8.91 (5.37, 13.7) |
| 15-21 | 184 | 124 | 60 | 32.6 (25.9, 39.9) |
| 22-28 | 231 | 106 | 125 | 54.1 (47.5, 60.7) |
| 29-35 | 416 | 135 | 281 | 67.5 (62.8, 72.0) |
| ≥35 | 238 | 61 | 177 | 74.4 (68.3, 79.8) |

**Footnotes**

For the calculation of positive predictive value, the blood slide result was considered as the gold-standard (reference) diagnosis.

^a^ SMC: seasonal malaria chemoprevention

^b^ RDT: rapid diagnostic test

**Supplementary Table 3.** Incidence of blood-slide confirmed clinical malaria by time since the most recent SMC treatment

|  | **SMC ^a^ plus placebo** | | **SMC plus azithromycin** | |  |  |
| --- | --- | --- | --- | --- | --- | --- |
|  |  |  |  |  |  |  |
| **Days since last SMC** | **No. of events (PYAR) ^b^** | **Events per 1000 person-years at risk (95% CI) ^c^** | **No. of events (PYAR)** | **Events per 1000 person-years at risk (95% CI)** | **Rate Ratio ^d^ (95% CI)** | **LRT ^e^**  **P-value** |
| 0-7 | 25 (2044.3) | 12.2 (8.24, 19.0) | 35 (2051.4) | 17.1 (12.3, 24.5) | 1.38 (0.83, 2.31) |  |
| 8-14 | 20 (2030.3) | 9.85 (6.46, 15.8) | 14 (2036.9) | 6.87 (4.15, 12.3) | 0.69 (0.35, 1.37) |  |
| 15-21 | 38 (1983.8) | 19.2 (14.1, 26.7) | 50 (1991) | 25.1 (19.0, 33.9) | 1.30 (0.85, 1.98) |  |
| 22-28 | 80 (1805.4) | 44.3 (35.8, 55.5) | 87 (1814.5) | 47.9 (38.8, 60.0) | 1.07 (0.79, 1.45) |  |
| 29-35 | 138 (701.8) | 196.6 (166.4, 234.0) | 179 (704.7) | 254.0 (219.0, 296.3) | 1.28 (1.02, 1.60) |  |
| >35 | 92 (498.8) | 184.5 (150.9, 228.0) | 92 (484.9) | 189.7 (154.6, 235.5) | 0.98 (0.73, 1.31) | 0.384 |

^a^ SMC: seasonal malaria chemoprevention;

^b^ PYAR, Person-years at risk;

^c^ CI: confidence interval;

^d^ For each time stratum, incidence rate was calculated as number of events divided by person-years at risk. Rate ratios compare SMC+Azithromycin versus SMC+placebo groups. Poisson regression models, with a gamma distributed random effect to account for the household randomisation and within-individual clustering of morbidity episodes. Models were adjusted for study country only.

^e^ Likelihood ratio test comparing models with and without an interaction between treatment group and time since treatment.

**Supplementary Figure 1**. Incidence of clinical malaria by study group among the sub-set of children with blood slide-confirmed malaria


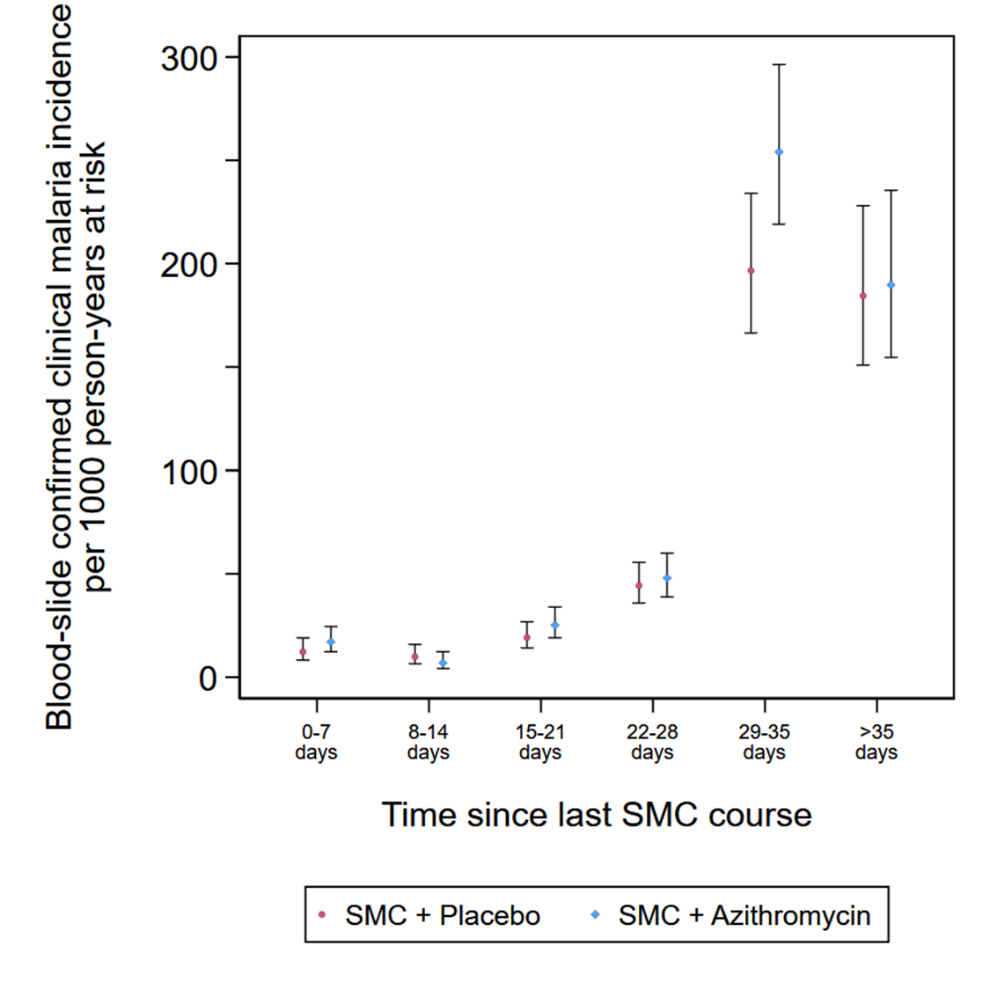


**Footnotes:**

For each time stratum, incidence rate was calculated as number of events divided by person-years at risk. Red and blue squares are point estimates, solid vertical lines indicate 95% confidence intervals.

SMC: seasonal malaria chemoprevention

**Supplementary Figure 2**. Protective efficacy of SMC+Azithromycin vs. SMC+Placebo among the sub-set of children with blood slide-confirmed malaria


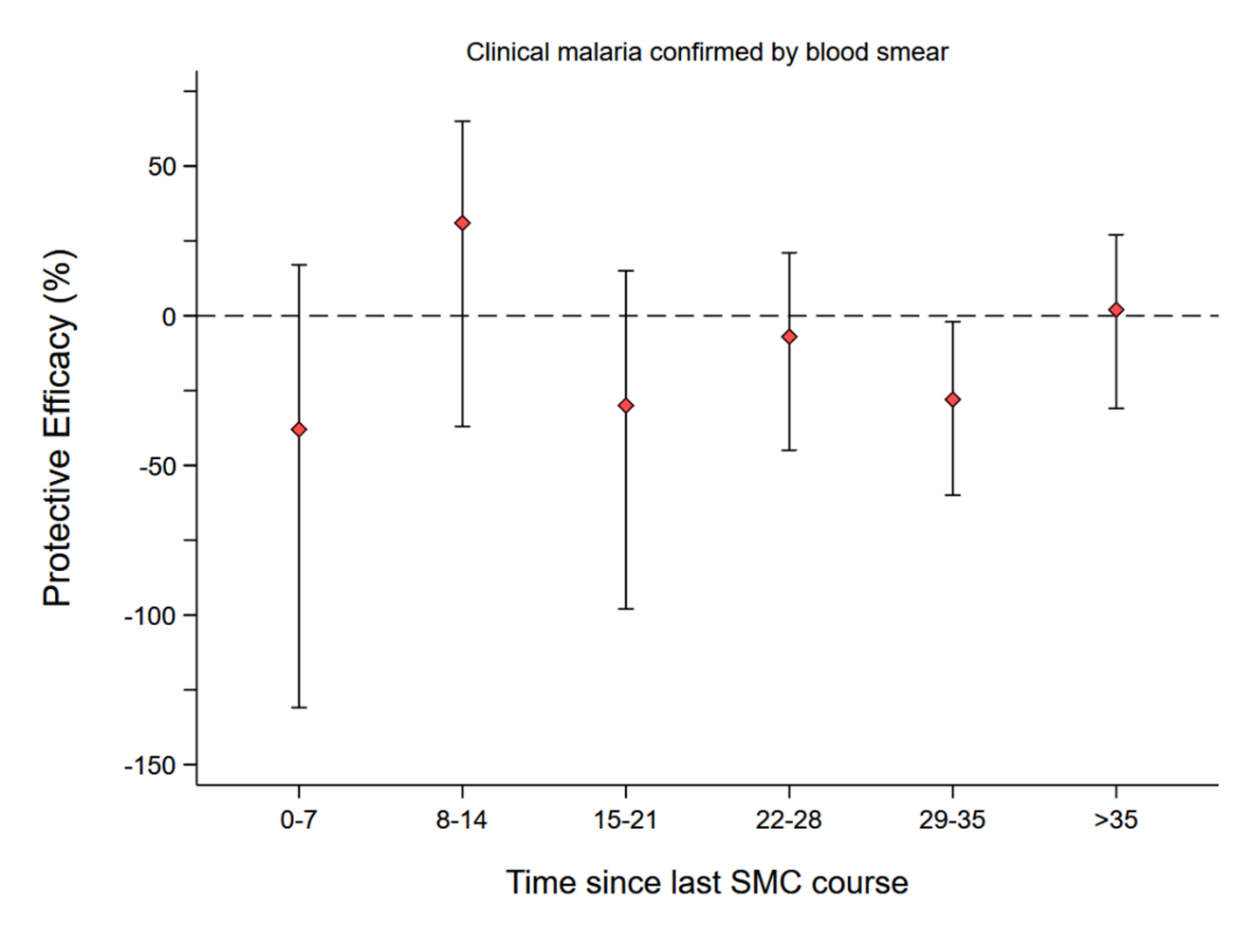


**Footnotes:**

Protective efficacy calculated as 100*(1-Rate ratio comparing SMC+Azithromycin / SMC+placebo groups), estimated from Poisson regression models, with a gamma distributed random effect to account for the household randomisation and within-individual clustering of morbidity episodes. Models were adjusted for study country only. Dashed horizontal line indicates no protective efficacy of azithromycin. For clarity of presentation the Y-axis is truncated at -100 for ALRI. The lower limit of the CIs for 29-35 days and >35 days extend to –156% and -188%, respectively.

SMC: seasonal malaria chemoprevention
